# The rate of electrical energy dissipation (power) and the RC constant unify all electroporation parameters

**DOI:** 10.1007/s13205-012-0105-1

**Published:** 2012-12-11

**Authors:** Paul F. Lurquin

**Affiliations:** School of Molecular Biosciences, Washington State University, c/o P.O. Box 369, Cannon Beach, OR 97110 USA

**Keywords:** Electroporation, RC constant, Electrical power dissipation, Unification of parameters

## Abstract

Electroporation parameters can be optimized by coupling RC constant values with the amount of electrical power dissipation in the electroporation medium. Electroporation efficiency increases more steeply with power at low power values.

Cell electropermeabilization (electroporation) is a seemingly complex function of electrical parameters such as voltage, capacitance, resistance, electrical pulse length, and electric field strength. It is shown here that successful conditions of electroporation can be estimated and standardized by computing and correlating the rate of electrical energy discharge (power) and pulse length values.

Electroporation is widely used to introduce molecules large and small into living cells. Pore formation in cell membranes is thought to occur via electric field stimulation of first, membrane compression and next, membrane breakdown in localized spots (Lurquin 1997). Molecules present in the surrounding medium are then able to diffuse through the temporary pores thus formed. Experiments with artificial liposomes support this model (Lurquin and Athanasiou 2000).

Since electropore formation is a purely physical phenomenon, properly defined electroporation conditions suitable for one type of cell membrane (bacterial, fungal, plant, and mammalian) may be applicable to all types, taking into account cell size. This was indeed shown to be the case many years ago (Lurquin 1997). Yet, current articles reporting electroporation parameters almost invariably provide a “cookbook” approach to setting up these parameters, as if the process were largely of a trial-and-error nature. These reports, while valuable, usually make no attempts to correlate electrical parameters (resistance, voltage, pulse length, capacitance, and electric field strength) with one another (e.g., ref. 3). In fact, we showed earlier that electroporation conditions can be standardized if they are based on the amount of electrical energy dissipated into the electroporation cell and not on the above electrical parameters considered separately (Lurquin 1997, 2002; Chen et al. 1998).

The importance of energy dissipation during electroporation was first established theoretically (Lurquin 1997), and soon demonstrated empirically using plant protoplasts (Chen et al. 1998). In addition to energy dissipation, the significance of the rate of energy dissipation was raised but not solved (Lurquin 1997). This issue is addressed in this paper. But first, it is necessary to review briefly the theoretical basis of electropore formation. As mentioned earlier, membrane breakdown in an electric field is dependent on cell size and is governed by the simplified Laplace equation *V* = 1.5 *r E,* where *V* is in the breakdown voltage (very approximately 1 V) and *r* is the cell radius in centimeters (Lurquin 1997). *E* is the applied electric field strength in V/cm. In practice, bacterial cells are porated around 12–16 kV/cm, microeukaryotes at about 1 kV/cm, and eukaryotic cells at about 0.3–0.7 kV/cm (Lurquin 1997). Large liposomes (2.5–20 μ in diameter) made of di-palmitoyl-phosphatidyl-choline or l-α-phosphatidyl-choline are efficiently electroporated at 1.5 kV/cm (Lurquin and Athanasiou 2000). Thus, the first rule of electroporation is to achieve membrane breakdown, which is dependent on cell size, not cell type (Lurquin 1997). Cell survival must be determined empirically in all cases, but is expected to be excellent within the limits given above.

For capacitor discharges, pulse length is determined by the RC constant (resistance × capacitance) expressed in seconds. We will see below that the pulse length also plays a role in electroporation efficiency. Electrical energy dissipation є = 0.5 C V^2^ is expressed in Joules (J) and is thus equal to 0.5 × capacitance (in μFarads) × voltage squared (in Volts). Electroporation efficiency is thus directly proportional to є, whose values typically range from about 80 J for *Escherichia coli* to about 25 J for yeast, to about 23 J for HeLa cells, to about 35 J for soybean (*Glycine max*) protoplasts (Lurquin 1997). More generally, for eukaryotic cells, adequate energy values for plant protoplasts are in the 30–50 J range while for mammalian cells the range is 10–30 J (Lurquin 1997). For example, the є value for HUVEC cells is 30.6 J, it is 15.6 J for SK-N-SH and CHO DG44 cells, and 11.25 J for K562 cells (calculated from Jordan et al. 2008).

What, then, is the importance of pulse duration (dictated by the RC constant) on the energy dissipation process and its effect on electroporation efficiency? This issue is solved here by re-analyzing and computing older energy data obtained with plant (*Asparagus officinalis*) protoplasts as reported in ref. (Chen et al. 1998). These authors noticed an effect of the RC constant on electroporation efficiency, but did not elaborate further. Electrical energy dissipation as a function of time is called electrical power (*P* = dє/d*t*) and is measured in J/s. Figure 1 shows that electroporation efficiency and pulse length at various power levels are clearly correlated. Each data point corresponds to quadruple experiments with the same high statistical significance as in ref. 4 (Chen et al. 1998) (also see legend of Fig. 1). Thus, for each pulse length, electroporation efficiency increases with power.

**Fig. 1:**
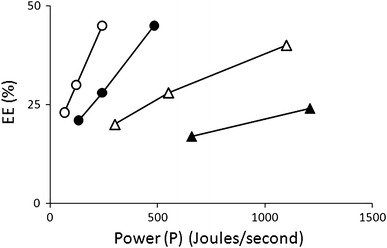
Electroporation efficiency (*EE*) of *Asparagus officinalis* protoplasts as a function of power (*P*) dissipation in the electroporation medium at various pulse lengths. *Open circles* RC = 100 ms, *closed circles* RC = 50 ms, *open triangles* RC = 22 ms, *closed triangles* RC = 10 ms. Data points were computed from energy and average electroporation efficiency values as reported in (Chen et al. 1998). All data points preserve the original statistical significance of electroporation efficiency based on regression analysis and LSD_0.05_ as in (Chen et al. 1998). The figure was created with assistance from Dr. Kathryn Gibson, School of Veterinary Medicine, St. George’s University, Grenada, West Indies

Two further conclusions can be drawn from these computations: (1) electroporation efficiency increases more rapidly with power at longer pulse times. This is in accordance with the known fact that longer pulse times decrease membrane breakdown voltage (Lurquin 1997), presumably increasing the number of electropores formed and possibly their stability. The correlation between electroporation efficiency and the RC constant can be quantified by calculating the former’s rate of increase (its slope) versus power at each value of RC; thereby, estimating (ΔEE/ΔP)_RC_ (Table 1). It can be seen that the increment in electroporation efficiency per unit power is proportional to the RC constant, being ten times higher at 100 ms than it is at 10 ms; (2) the same electroporation efficiency can be achieved at widely different power values, depending on pulse length. For example, Fig. 1 shows that ca. 40–45 % electroporation efficiency is achieved at *P* = 242 J/s if RC = 100 ms, *P* = 484 J/s with RC = 50 ms, *P* = 1,100 J/s with RC = 22 ms. But only about 24 % electroporation efficiency is reached at *P* = 1,210 J/s with RC = 10 ms. This also means that optimization of electroporation conditions can be done within a much narrower range of P values at long pulse times. Interestingly, pulse length from 100 to 10 ms had no effect on cell viability (Chen et al. 1998). The correlation of electroporation efficiency and power at *t* = 10 ms has only two data points. It is shown for completeness. It should be noted that, by virtue of the cross-sectional nature of the present re-analysis, the slope at RC = 10 ms retains the high statistical significance of the data points as provided in (Chen et al. 1998).

**Table 1:** Correlation between the rate of increase of electroporation efficiency (ΔEE) and power (ΔP) at four values of RC

RC (ms)	(ΔEE/ΔP)_RC_ (% EE efficiency increment/J/s)
10	0.02
22	0.05
50	0.10
100	0.21

Data calculated from Fig. 1

*ΔEE* electroporation efficiency increment, *ΔP* power increment

Typical *P* values for a variety of cell lines are as follows: about 15,000 J/s (at RC = 4.8 ms) for *E. coli*, 5,000 J/s (at RC = 4.5 ms) for yeast, 760 J/s (at RC = 45 ms) for soybean protoplasts, and 643 J/s (at RC = 35 ms) for He La cells (Lurquin 1997). For large liposomes, efficient poration is seen at *P* = 1,700 J/s (at RC = 12 ms) (Lurquin and Athanasiou 2000). Since energy factors are the same for capacitor discharges and square pulses (albeit calculated differently) (Lurquin 1997; Lurquin 2002), it is likely that power correlation with pulse length leads to similar electroporation efficiency with both techniques. It is suggested that companies that build electroporation units include energy and power values in the settings on their instruments. This would streamline the search for best electroporation conditions.
